# Design and Implementation of a Novel Tilt Sensor Based on the Principle of Variable Reluctance

**DOI:** 10.3390/s19235228

**Published:** 2019-11-28

**Authors:** Lei Guo, Lishuai Zhang, Yuan Song, Liang Zhao, Qiancheng Zhao

**Affiliations:** School of Automation, Beijing University of Posts and Telecommunications, Beijing 100876, China; 15604016232@163.com (L.Z.); songyuan@bupt.edu.cn (Y.S.); zhao_liang@bupt.edu.cn (L.Z.); zhaoqiancheng11@163.com (Q.Z.)

**Keywords:** tilt sensor, damping plate, eccentric structure, variable reluctance, translational acceleration restraint, dynamic tilt angle measurement

## Abstract

Tilt angle measurement in dynamic systems is problematic because the rotation of the measured platform is coupled with translation. Therefore, when some sensors are applied in dynamic systems, their output signals are often submerged in the noise signals generated by translation. To enhance the ability of tilt sensors to resist translational noise, a dynamic tilt sensor is proposed based on the principle of variable reluctance from the perspective of sensor structure. The eccentric structure of the sensor constructed with a shell, liquid, and internal damping plate was designed according to the principles of mechanics. The characteristic of translational acceleration restraint determined by the sensor structure was established theoretically. In addition, the magnetic circuit of the sensor was analyzed to illustrate the sensor’s working principles. A Clapp oscillator circuit was designed to convert mechanical motion into a measureable electrical signal. A method to determine the sensor’s direction of rotation is proposed. A waveform conversion circuit was designed to convert the sine wave output of the Clapp oscillator to a square wave, and a square-wave frequency measurement circuit was designed based on the C8051 micro-control unit. A translation–rotation experimental hardware platform was constructed. The data acquisition program was designed on a PC platform, and the translation–rotation experiments were conducted with an MTi attitude measurement unit as a reference. The validity of the tilt angle measurements and the effect of the translational acceleration restraint of the sensor were verified by the experimental data. The theoretical results obtained were consistent with the experimental data, verifying the validity of the theoretical analysis and experimental devices employed. A measurement range of −180 to 180° was achieved.

## 1. Introduction

In all kinds of feedback control systems, the importance of the sensor as the main component of the feedback link is self-evident. Its output signal is often the important basis for the control system to make decisions. Distortion of the feedback signal can bring disastrous consequences. In the research of robot balance control, we need to measure the roll angle of the robot as the feedback signal of the control system in order to keep the robot’s posture balanced in the process of motion. However, we find that translational motion seriously interferes with the output value of the sensor. One existing dip sensor is based on the principle of capacitance [1]. After a thorough investigation of all major nonlinear effects, researchers designed an accurate system-level model of the sensor, which is capable of handling signal offset and amplitude variations. A high–accuracy, easy–to–fabricate capacitive angle transducer that provides digital output linearly proportional to the angle being sensed in the range 0 to 360° is presented in [2]. Ha et al. presented design optimization and validation of a two-axis cylindrical capacitive tilt angle sensor. The proposed sensor includes five electrodes symmetrically arranged surrounding an air–liquid two–phase cylindrical tube, forming two pairs of electrodes for roll and pitch angle measurement [3]. Chen et al. adopted differential capacitance detection and made use of the approximately proportional relationship between the capacitance difference and the rotor deflection angle to construct a new angle sensor [4].

Another type of inclination sensor was designed based on the principle of a gas pendulum. Zhang proposed that the natural convention gas in the closed cavity has the characteristics of a pendulum. Compared with solid and liquid pendulum inertial sensors, gas pendulum inertial sensors have the advantages of strong shock resistance and short response time [5,6]. In [7], the pendulum characteristic of natural convection gas within a 3D spherical closed cavity is discussed. By using the finite element method, the flow and temperature field caused by the heat source within the 3D spherical closed cavity under different tilted conditions were calculated.

Some tilt sensors are designed based on acceleration sensors. An inclination sensor based on a micro-electromechanical system (MEMS) accelerometer had high accuracy is high but was affected by vibration, impact, temperature and other factors [8]. In [9], the authors designed and implemented a novel algorithm for an embedded microcontroller such that the system was capable of transferring the raw data of the accelerometer from the motion domain to the angular domain in-line, in order to provide the inclination or tilt angle information alone in real time. However, the angle calculation was very complicated [9]. In another paper, a new type of digital inclination meter was studied. The signal processing of a MEMS accelerometer was divided into adjustment, sampling, conversion, and temperature compensation, and then the tilt angle was calculated [10].

Many kinds of tilt sensors designed based on optical principles have been developed in recent years. For a fiber Bragg grating inclination sensor (FBG), the experiment showed that although its measurement accuracy was very high, it had high requirements in terms of the temperature of the measurement environment [11,12,13,14], and the measurement range was usually relatively small [15].

The absence of a torque state has been established for an ideal liquid in a ball cavity [16,17], and a tilt sensor making use of the effect of liquid damping was presented [18], although the sensor had a major drawback in that its measurement range was very small. 

We can make the following summary: Some sensors are only suitable for static environments and are sensitive to both rotation and translational motion. For some sensors, their measurement range is limited. For example, the CXTA-01 sensor can only maintain good measurement accuracy within a limited range. Temperature drift has a big effect on some sensors, such as the MPU6050 sensor. Some tilt sensors, such as fiber optic gyroscopes, can be applied in dynamic systems, but they are relatively expensive. Therefore, designing a low-cost dynamic angle sensor is urgent for practical applications. Thus we propose a new type of dynamic inclination sensor with an eccentric structure based on the principle of solid pendulum with liquid damping to improve the characteristics of the sensor.

This paper presents a sensor with an eccentric structure built with a shell, liquid, and internal damping plate. The sensor’s measurement range covers a full 360°. Owing to the sensor’s eccentric structure, when it rotates to a certain angle, the reluctance is changed, and the inductance of an inductance coil is correspondingly varied, which is used to convert the mechanical motion of the sensor into a measurable electrical signal. Experimental studies on an experimental platform were performed to verify the validity of the proposed sensor.

This paper makes several contributions to the literature:

(1) It is the first time the novel structure of a sensor with a measurement range of 360° is presented. According to the requirement, sensors with this structure can be made very large or very small.
(2) In order to reduce the influence of translation noise, the sensor is filled with oil. A damping board fixed in the sensor, with the structure of a buoyancy center moving up from the geometric center and the gravity center moving down from the geometric center is proposed.
(3) A scheme of non-electricity conversion is proposed. Mechanical motion such as roll angle is transformed into the variation of reluctance. The variation of reluctance causes the change of inductance value of the sensor coil. The change of inductance leads to the change of output frequency of the clapper oscillator circuit.

## 2. Sensor Structure Design 

**Figure 1:**
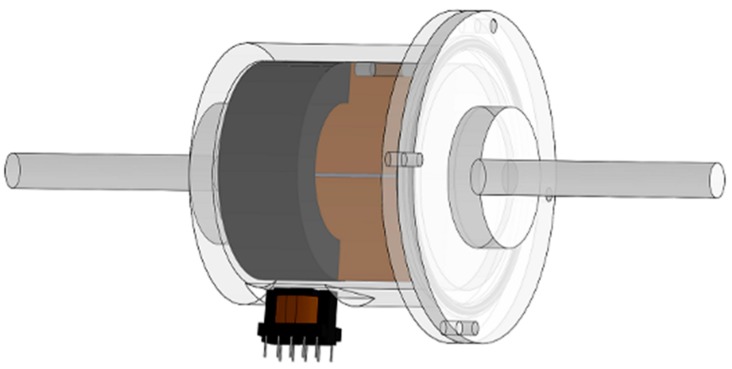
Illustration of proposed sensor.

**Figure 2:**
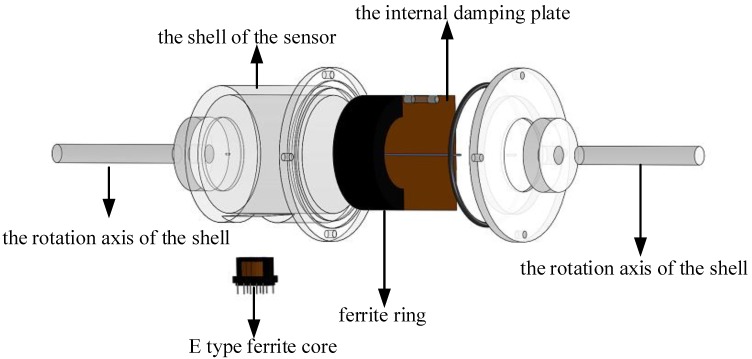
Assembly and exploded diagram of proposed sensor.

Illustrations of the sensor designed in the present study are shown in Figure 1 and Figure 2. To overcome the limited measurement range discussed with regard to the tilt sensor in [18], the following two improvements were adopted: First, the I-type ferrite core that was fixed on the internal damping plate of the sensor in [18] was replaced by a ferrite ring. Second, the axis of the ferrite ring does not coincide with the rotational axis of the internal damping plate, although the two axes are parallel. 

**Figure 3:**
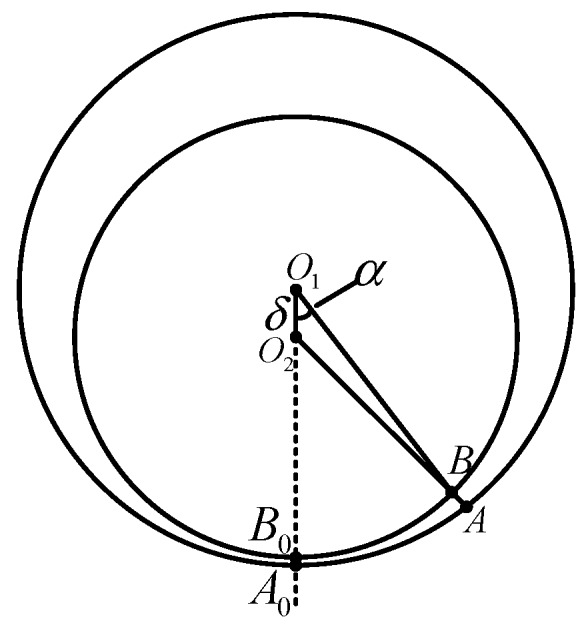
Cross-sectional view of sensor perpendicular to ferrite ring and internal damping plate rotational axes.

Figure 3 presents a cross-sectional view of the sensor perpendicular to the ferrite ring and the internal damping plate rotational axes. The circle with *O*_1_ as its center represents the shell of the sensor, and the circle with *O*_2_ as its center represents the ferrite ring. The distance between *O*_1_ and *O*_2_ is denoted by *δ*. When the sensor has a certain tilt angle *α*, the E-type ferrite core is fixed at point *A*. The cross point of radius *O*_1_*A*_0_ with circle *O*_2_ is at *B*_0_. Point *A* will rotate with the shell from *A* to *A*_0_, and the cross point of radius *O*_1_*A* with circle *O*_2_ is at *B*. Therefore, the distance between the ferrite ring and the shell can be expressed with angle *α*. 

## 3. Theoretical Analysis 

### 3.1. Sensor Analysis of Gas Gap in the Sensor

The structure of the sensor has the following characteristics. Its performance is associated with distance *O*_1_*O*_2_, which is the linear eccentricity, as shown in Figure 3. This indicates that the value of *O*_1_*A*_0_ or *O*_2_*B*_0_ has no bearing on the performance of the sensor. Therefore, this structure allows the sensor to be as large or small as desired.

Let *d* denote the distance of the gas gap *AB* shown in Figure 3. Let *R* denote the cylindrical cavity radius of the sensor, and let *r* denote the outer radius of the ferrite ring. 

As such, it is shown in Figure 3 that *R* = *O*_1_*A*, *r = O*_2_*B*, and *δ* = *O*_1_*O*_2_.

Let *x* denote the distance *O_1_B*. According to the law of cosines for the triangle *O*_1_*O*_2_*B*, we can propose the following equation:

(1) $$x^{2}+\delta^{2}-2\delta x\cos\alpha =r^{2}$$

This equation can be represented as follows:

(2) $$x^{2}-2\delta x\cos\alpha +\delta^{2}-r^{2}=0$$

When *δ*, *r*, and re known variables, the above equation is a quadratic equation, and its solution is as follows:

(3) $$x=\delta \cos\alpha \pm \sqrt{\delta^{2}\cos^{2}\alpha +r^{2}-\delta^{2}}$$

In the design of the sensor, we assume a small eccentricity *δ*, which provides the following relationship, as shown in Figure 3:

(4) δ≪r<R

The following relationship obviously follows:

(5) $$r^{2}-\delta^{2}>0$$

Moreover, $\delta^{2}\cos^{2}\alpha \geq 0$.

As such, the following relationship is obtained:

(6) $$\delta^{2}\cos^{2}\alpha +r^{2}-\delta^{2}>0$$

It is proved that Equation (2) has two distinct real roots given by Equation (3). Some circumstances were considered to check the above two roots. It is not difficult to determine that the following is a reasonable root:

(7) $$x=\delta \cos\alpha +\sqrt{\delta^{2}\cos^{2}\alpha +r^{2}-\delta^{2}}$$

Then, the distance of the gas gap *AB* shown in Figure 3 can be presented as follows:

(8) $$d=\left|AB\right|=\left|AO_{1}\right|-\left|BO_{1}\right|=R-x=R-\delta \cos\alpha -\sqrt{\delta^{2}\cos^{2}\alpha +r^{2}-\delta^{2}}$$

Therefore, *d* can be presented as follows:

(9) $$d=R-\delta \cos\alpha -\sqrt{r^{2}-\delta^{2}+\delta^{2}\cos^{2}\alpha }$$

### 3.2. Reluctance Analysis of the Sensor

**Figure 4:**
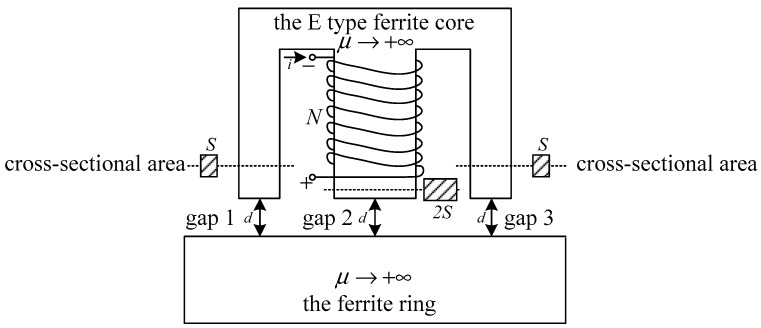
Reluctance analysis of sensor.

**Figure 5:**
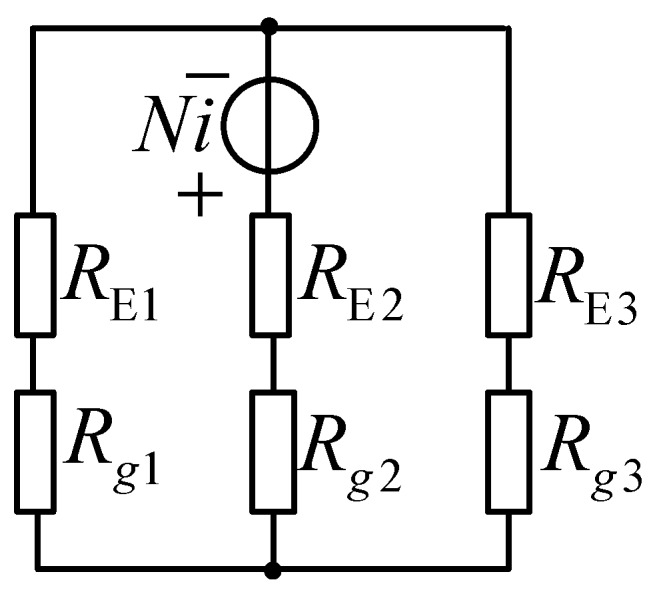
Equivalent magnetic circuit.

**Figure 6:**
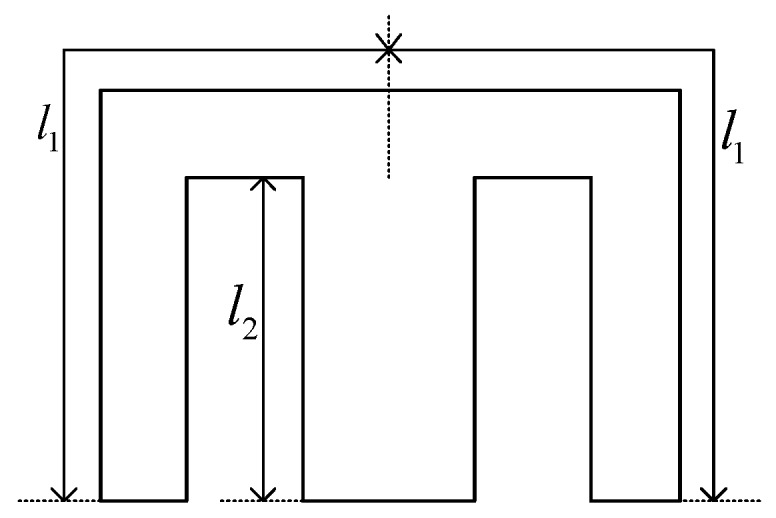
Geometric parameters of magnetic circuit of E-type ferrite core.

As shown in Figure 4, the middle core element of the E-type ferrite core has *N* turns. Three gaps of the same distance *d* lie between the ends of the E-type ferrite core and the ferrite ring. The cross-sectional areas of gap 1 and gap 3 are equivalent, and are denoted by *S*. Similarly, the cross-sectional area of gap 2 is denoted as 2*S* according to the characteristics of the E-type ferrite core. Finally, it is assumed that the permeability μ of the ferrite is infinite. As such, an equivalent magnetic circuit of Figure 4 can be presented, as shown in Figure 5. Here, *R_E1_*, *R_E2_*, and *R_E3_* denote the reluctance values of the ferrite core elements in the E-type core, and *R_g_*_1_, *R_g_*_2_, and *R_g_*_3_ denote the reluctance of gaps 1, 2, and 3, respectively. The geometric parameters of the E-type ferrite core are shown in Figure 6, where *l*_1_ and *l*_2_ denote the length of the magnetic circuit. According to the characteristics of the E-type ferrite core, the flux of a magnet of length *l*_1_ is half that of a magnetic with length *l*_2_.

From the above considerations, the following relationship can be obtained:

(10) $$R_{E1}=R_{E3}=\frac{l_{1}}{\mu S},\;R_{E2}=\frac{l_{2}}{2\mu S}$$

Let the permeability of a vacuum be defined as $\mu_{0}$. Without regard for the magnetic field boundary conditions, the following relationship can be obtained:

(11) $$R_{g1}=R_{g3}\approx \frac{d}{\mu_{0}S},\;R_{g2}=\frac{d}{2\mu_{0}S}$$

Because $\mu \gg \mu_{0}$, the following relationship can be obtained:

(12) $$R_{g1}=R_{g3}\gg R_{E1}=R_{E3},\;R_{g2}\gg R_{E2}$$

**Figure 7:**
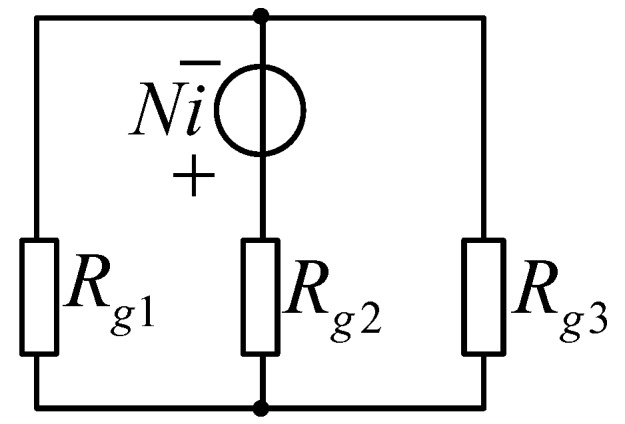
Simplified equivalent magnetic circuit.

Therefore, the equivalent magnetic circuit shown in Figure 5 can be simplified as given in Figure 7.

Denoting the flux through the middle core element as ϕ and the total magnetic reluctance as $R_{m}$, the flux through the main magnet can be presented as follows:

(13) $$\phi =\frac{Ni}{R_{m}}$$

The inductance of the coil with *N* turns on the middle core element of the E-type ferrite core is proposed as follows:

(14) $$L=\frac{N\phi }{i}$$

Equation (13) can be substituted into Equation (14), and the following relationship obtained:

(15) $$L=\frac{N^{2}}{R_{m}}$$

In addition, *R_m_* can be presented as follows, according to Figure 7:

(16) $$R_{m}=R_{g1}||R_{g3}+R_{g2}$$

**Figure 8:**
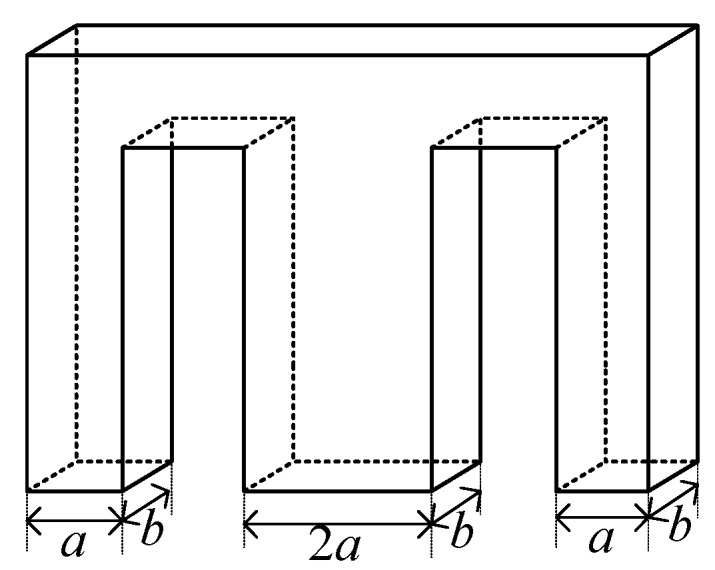
Geometric parameters of gaps between E-type ferrite core and ferrite ring.

However, Equation (16) is too idealized to describe the gap reluctance precisely, and a more accurate expression is required. The geometric parameters of the gap between the E-type ferrite core and the ferrite ring are shown in Figure 8. Because *r* is much larger than thickness *b* of the E-type ferrite core, the following inequality holds:

(17) r≫b

**Figure 9:**
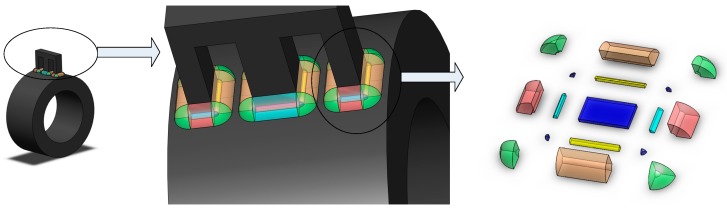
Analysis of gap reluctance between E-type ferrite and ferrite ring cores.

Accordingly, we can reasonably approximate that the outer cylindrical surface of the ring-type ferrite core is a plane with respect to the E-type ferrite core element surfaces. Considering the edge effects of the magnetic field in the gap, the distribution of reluctance in the gaps between the E-type ferrite and ferrite ring cores can be illustrated as shown in Figure 9. The permeance of all portions of the gaps shown in Figure 9 is presented in Table A1 in Appendix A.

The total reluctance of the gaps can be represented as follows, for which the derivation processes of reluctance $R_{g1}$, $R_{g2}$, and $R_{g3}$ are shown in Appendix A.

(18) $$R_{m}=0.5R_{g1}+R_{g2}=\frac{1}{2\mu_{0}(\frac{A}{d}+3.232d+B_{1})}+\frac{1}{\mu_{0}(\frac{2A}{d}+3.232d+B_{2})}$$

Here, $B_{1}=a(1+\frac{4}{\pi }\ln(2))+b(1+\frac{4}{\pi }\ln(2))$,$B_{2}=a(2+\frac{8}{\pi }\ln(2))+b(1+\frac{4}{\pi }\ln(2))$, and A=ab.

Substituting Equation (18) into Equation (15) provides the following relationship:

(19) $$L=q_{1}(d)$$

Function $q_{1}$ indicates that exact values of *L* are determined by *d*.

Substituting Equation (9) into Equation (19) yields the following relationship:

(20) $$L=q_{2}(\alpha )$$

Function $q_{2}$ indicates that exact values of *L* are determined by α. Therefore, measurements of *L* can provide the value of α to convert the mechanical motion into a measureable electrical signal.

### 3.3. Design of the Clapp Oscillator

**Figure 10:**
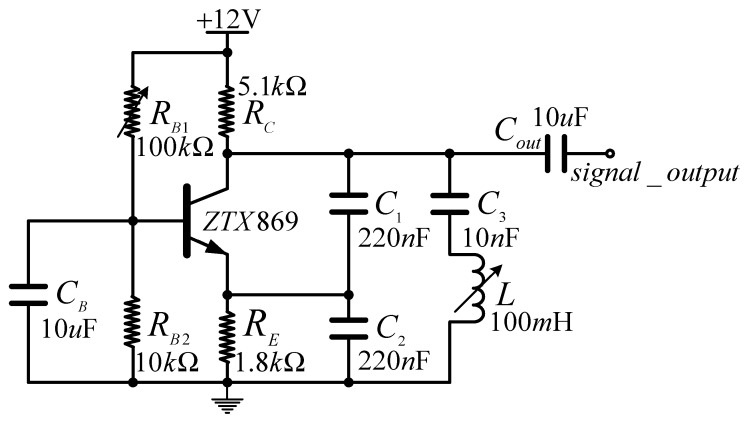
Design of the Clapp oscillator.

To measure the values of *L*, we designed the sine wave oscillator circuit shown in Figure 10, known as a Clapp oscillator. The design is based on the expectation of low oscillation frequency (3–10 kHz) and the measurement of fairly large inductor values (30–67 mH). Therefore, it may be appropriate to assume large capacitance values for capacitors $C_{1}$ and $C_{2}$, such as 220 nF, and the capacitance of $C_{3}$ was set as 10 nF. As shown in Figure 10, $R_{B1}$ is an adjustable resistance for obtaining the appropriate quiescent operating point of the BJT ZTX869 transistor.

The variable inductance shown in Figure 10 is an abstraction of the inductance of the middle core element of the E-type ferrite core with *N* turns, reflecting that when the sensor is rotated to a certain angle, the inductance value of the coil is changed according to Equation (20). The function of the circuit is to transform the values of *L* into an oscillation frequency $f_{out}$ for measurement with a digital system according to the well-known frequency relationship for the Clapp oscillator presented as follows:

(21) $$f_{out}\approx \frac{1}{2\pi \sqrt{LC_{3}}}$$

Substituting Equation (20) into Equation (21), the following relationship can be proposed.

(22) $$f_{out}=q_{3}(\alpha )$$

Function $q_{3}$ indicates that exact values of $f_{out}$ are determined by α.

### 3.4. Design of the Waveform Conversion Circuit

**Figure 11:**
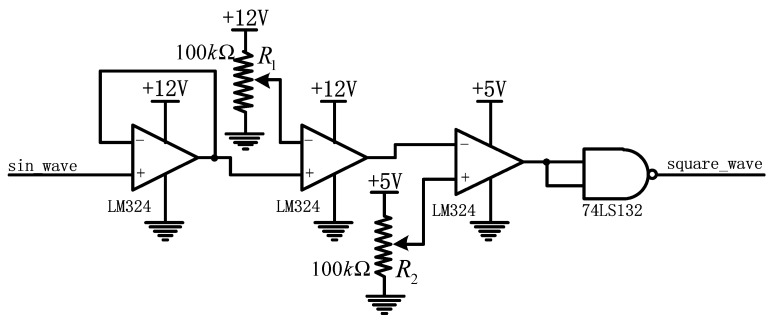
Design of the waveform conversion circuit.

To achieve the goal of measuring $f_{out}$ for the sine wave output of the Clapp oscillator with the C8051 micro-control unit (MCU), a waveform conversion circuit was designed, as shown in Figure 11. The first LM324 operational amplifier (at left) constitutes a voltage follower to obtain the sinusoidal signal. The second LM324 constitutes a single threshold voltage comparator for converting the sinusoidal signal to a square wave signal with an amplitude of 12 V. The third LM324 is also a single comparator threshold voltage comparator, but its supply voltage is 5 V to convert its input to a square wave signal of 5 V. Finally, after shaping this square wave with the 74LS132 Schmitt trigger, a perfect square wave is obtained. This square wave is used as an input signal of the frequency meter to obtain an accurate measurement of $f_{out}$.

### 3.5. Analysis of the Sensor’s Direction of Rotation

As a result of the symmetric structure of the sensor, the frequency of the wave measured from the coil is the same whether the sensor rotates to an angle in a clockwise or counterclockwise direction. To judge the direction of rotation, a second coil must be fixed on the sensor at an interval of 90° from the first, where the first coil is defined as the main coil and the second is defined as the assistant coil. The direction of rotation can be determined by comparing the frequencies of the waves measured from the two coils under the known condition of a 90° interval between the two coils. The counterclockwise direction is defined as the positive direction, making the clockwise direction negative. When the two-coil sensor rotates to an arbitrary angle α, the frequency $f_{m}$ of the wave measured from the main coil is given as follows:

(23) $$f_{m}=q_{3}(\alpha )$$

Simultaneously, the frequency $f_{v}$ of the wave measured from the assistant coil is defined as follows:

(24) $$f_{v}=q_{3}(\alpha +90^{\circ })$$

**Figure 12:**
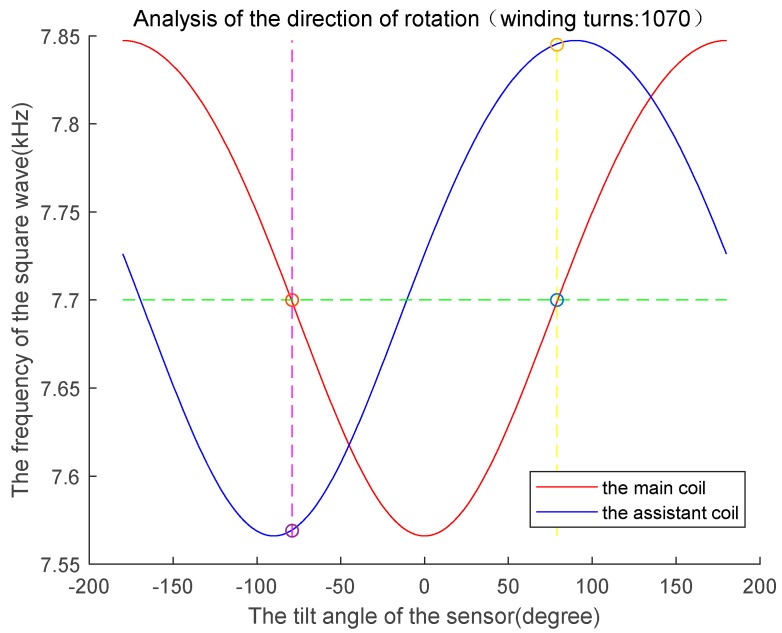
Analysis of sensor’s direction of rotation.

The rotation angle of the sensor can be obtained from Equation (23), but its inverse function is difficult to express. To simplify the rotational direction evaluation process, the angle can be determined according to two steps: (1) measure $f_{m}$ and $f_{v}$, and (2) evaluate the angles of rotation corresponding to the frequencies by searching a table containing the theoretical calculation results of Equations (23) and (24). For example, the sensor rotation angle can be determined by the following steps. First, construct a 1 × 361 array that begins from −180° and ends at 180° with steps of 1°. Second, two 1 × 361 arrays for the dependent variables $f_{m}$ and $f_{v}$ by computing Equations (23) and (24) with every member of the array in the first step. Third, construct an array $f_{m-\alpha }[2][361]$ and an array $f_{v-\alpha }[2][361]$ with the degrees and frequencies of the main coil and assistant coil corresponding to each degree, respectively. Finally, plot two frequency-degree curves in MATLAB with the arrays constructed in step 3. The curves of the sensor rotation simulation in Figure 12 were obtained for *N* = 1070 for both the main and assistant coils. The blue frequency-degree curve in the figure was obtained using array $f_{m-\alpha }[2][361]$ to represent the relationship between the frequencies and degrees of the main coil, and the red curve was obtained using array $f_{v-\alpha }[2][361]$ to represent that relationship of the assistant coil.

When the sensor is rotated to an arbitrary angle, the frequencies $f_{m}$ and $f_{v}$ are measured from the main and assistant coils separately. We drew a horizontal dotted green line in Figure 12 whose ordinate is the frequency $f_{m}$. Two intersection points are observed where the green line crosses the red theoretical curve in the figure. We define the two intersection points as $p_{1}$ and $p_{2}$. Obviously, $p_{1}$ and $p_{2}$ have the same ordinate, but the abscissas of the two points reflect the relationship $\alpha_{p_{1}}=-\alpha_{p_{2}}$. The actual rotation angle, which may be either abscissa $\alpha_{p_{1}}$ or $\alpha_{p_{2}}$, can be determined from the frequency $f_{v}$. We drew vertical dotted yellow and violet lines passing through the points $p_{1}$ and $p_{2}$, respectively. Two points of intersection with the blue theoretical curve are observed, which we define as points $p_{3}$ and $p_{4}$, respectively. Obviously, $p_{3}$ and $p_{4}$ have different ordinates. To determine the direction of the actual rotation angle, we compared the ordinates of $p_{3}$ and $p_{4}$ with the frequency $f_{v}$, and chose the ordinate of $p_{3}$ or $p_{4}$ (i.e., $f_{p_{3}}$ or $f_{p_{4}}$) lying closest to the frequency $f_{v}$ to determine the rotation angle of the sensor. The closest one was chosen because of errors in the measurement. For example, if the frequency $f_{p_{3}}$ is closer to the frequency $f_{v}$, the sensor rotation angle is $\alpha_{p_{1}}$. Similarly, if the frequency $f_{p_{4}}$ is closer to the frequency $f_{v}$, the sensor rotation angle is $\alpha_{p_{2}}$.

The method for determining the direction of sensor rotation can be summarized as follows:

Step 1. Search for angles $\alpha_{p_{1}}$ and $\alpha_{p_{2}}$ in the frequency-degree theoretical table $f_{m-\alpha }[2][361]$ according to the frequency $f_{m}$, which is measured from the main coil.

Step 2. Search for the frequencies $f_{m}$ and $f_{v}$ in the degree-frequency table $f_{v-\alpha }[2][361]$ according to angles $\alpha_{p_{1}}$ and $\alpha_{p_{2}}$, respectively.

Step 3. Choose the frequency closest to the frequency $f_{v}$.

### 3.6. Theoretical Analysis of the Sensor’s Dynamic Characteristics

**Figure 13:**
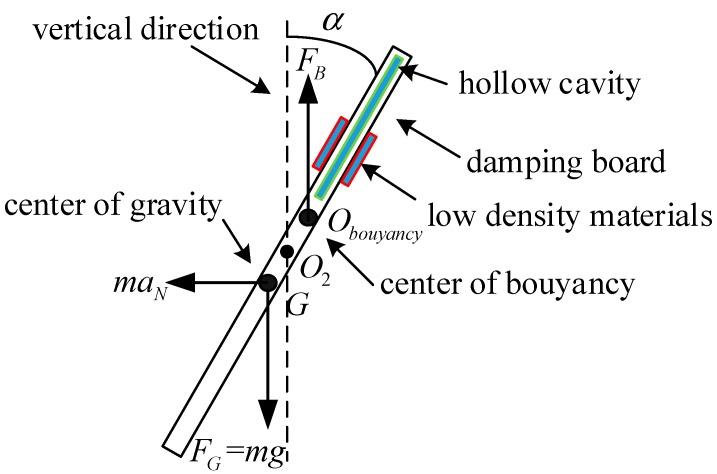
Analysis of forces on the damping board.

The area represented by the green line in Figure 13 is the hollow cavity in the damping board. For that reason, the density of the damping plate is not uniform. Also, the center of gravity (COG) of the damping board will be moved down from its geometric center. By adding the symmetrical red low-density materials on the damping board, its floating center can be moved up from the geometric center.

Figure 13 presents an analysis of the forces on the damping board when the angle between the damping board and the vertical direction is α and the translational acceleration of the sensor is given as $a_{N}$. In the figure, point *G* is the center of gravity the damping board, point $O_{2}$ denotes the fixed center of the board, and point $O_{bouyancy}$ is the buoyancy center of the board. The distance between *G* and $O_{2}$ is defined as $O_{2}G=l_{G}$, and the distance between $O_{bouyancy}$ and $O_{2}$ is $O_{2}O_{bouyancy}=l_{B}$. ρ is the density of the oil filled in the sensor, g denotes the acceleration due to gravity, and *V*_1_ indicates the volume of the damping board. *V*_2_ indicates the volume of the ferrite ring. The volume of the sensor’s rotor is *V* = *V*_1_ + *V*_2_. When the translational acceleration of the sensor is $a_{N}$, the inertial force on the damping board is $f_{N}=m_{r}a_{N}$. The forces of gravity and buoyancy on the damping board are given as $F_{G}=m_{r}g$ and $F_{B}=\rho gV$, respectively. The inner diameter of the ferrite ring is defined as $r_{0}$, and the length of the damping board is defined as *l*. We define the mass of the damping board and the ferrite ring as $m_{1}$ and $m_{2}$, respectively, indicating that the mass of the sensor’s rotor is $m_{r}=m_{1}+m_{2}$ (kg). The area represented by the green line in Figure 13 is the hollow cavity in the damping board. For that reason, the density of the damping plate is not uniform, and the center of gravity of the damping board will move down from the geometric center. By adding the symmetrical red low-density materials on the damping board, its floating center can be moved up from the geometric center.

Based on those definitions, the torque produced by buoyancy force is:

(25) $$M_{B}=\rho gVl_{B}\sin\alpha$$

The torque owing to gravity is:

(26) $$M_{G}=m_{r}gl_{G}\sin\alpha$$

The torque produced by the translational acceleration is:

(27) $$M_{N}=m_{r}a_{N}l_{G}\cos\alpha$$

Therefore, the equation reflecting the conservation of angular momentum can be represented as:

(28) $$J\frac{d^{2}\alpha }{dt^{2}}=m_{r}a_{N}l_{G}\cos\alpha -\rho gVl_{B}\sin\alpha -m_{r}gl_{G}\sin\alpha$$

In which, the sum of the rotational inertia of the damping boardand the ferrite ringis $J=m_{1}(\frac{1}{12}l^{2}+\delta^{2})+m_{2}(\frac{r^{2}+r_{0}{}^{2}}{2}+\delta^{2})$.

In the Equation (28), the translational acceleration is presented as follows:

(29) $$a_{N}=\frac{2}{19}\sin(2\pi ft).$$

**Figure 14:**
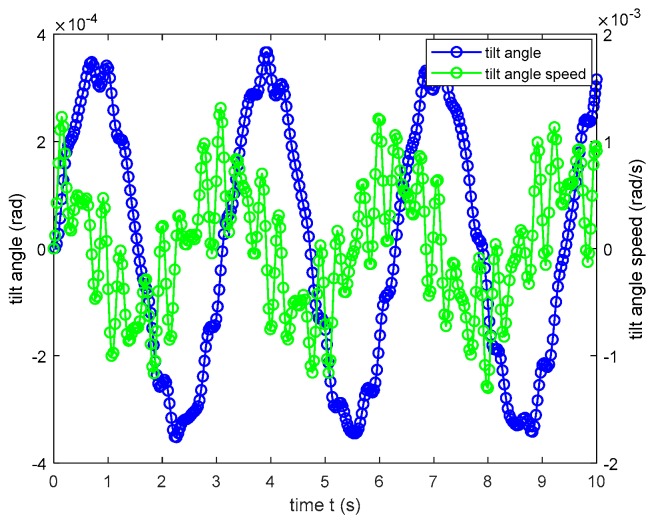
Numerical solution of Equation (28).

For initial conditions t=0 and α=0, the numerical solution of the above differential equation shown in Figure 14 can be computed using MATLAB.

In Figure 14, the blue curve (with blue markers) represents the sensor rotational degree, and the green curve (with green markers) represents the angular velocity of the damping board. The figure indicates that the maximum rotational degree and angular velocity of the damping board are 4 × 10^−4^ rad (0.0458°) and 3 × 10^−4^ rad/s (0.0344°/s), respectively. However, the dynamical model employed ignored the viscous resistance of the lubricating oil that fills the sensor chamber. Therefore, when accounting for viscous resistance, the rotation angle and angular velocity would be smaller and attenuate more rapidly than indicated in Figure 14. The preceding analysis verifies that translational acceleration has little influence on sensor rotation angle measurement.

### 3.7. Design of the Frequency Meter

The frequency of the square wave can be measured using the C8051 MCU. Prior to initiating the MCU, the environment must first be initialized. The initialization includes turning off the watch-dog, enabling the programmable counter array (PCA), setting the clock source of the PCA, and so on. The system clock is then established as the PCA counter’s clock, and the PCA module is set to function under the square wave positive edge-triggered capture mode.

Frequency is measured by a mode of inquiry that introduces error into the measurement. To reduce the error of a single measurement, we choose the mean filtering method to process the frequency of the square wave, which is counted by system clock cycles (the range of mean filtering is *K*, which is set to 20 in the experiment). The cycle of the system clock is measured by the PCA module. We set a cyclic sampling counter *i* in the program for mean filtering, which is initialized with a value of 1. Each time we obtain a cycle *T* of the square wave, *T* is accumulated to *avg_T*. When *i* = *K*, the mean of the square wave’s cycle is calculated as *avg_T/K*. If we suppose *avg_T* = z, *T* can be represented as:

(30) $$T=z\cdot \frac{1}{f_{osc}}=\frac{1}{f_{measure}}$$

where $f_{m\mathrm{ea}sure}$ is the pending measured frequency, which can be presented as follows:

(31) $$f_{m\mathrm{ea}sure}=avg\_F=\frac{1}{T}=\frac{f_{osc}}{z}$$

By the preceding equations, we can convert *avg_T* to frequency *avg_F*, convert *avg_F* to ASCII code, and output the data by a serial port.

**Figure 15:**
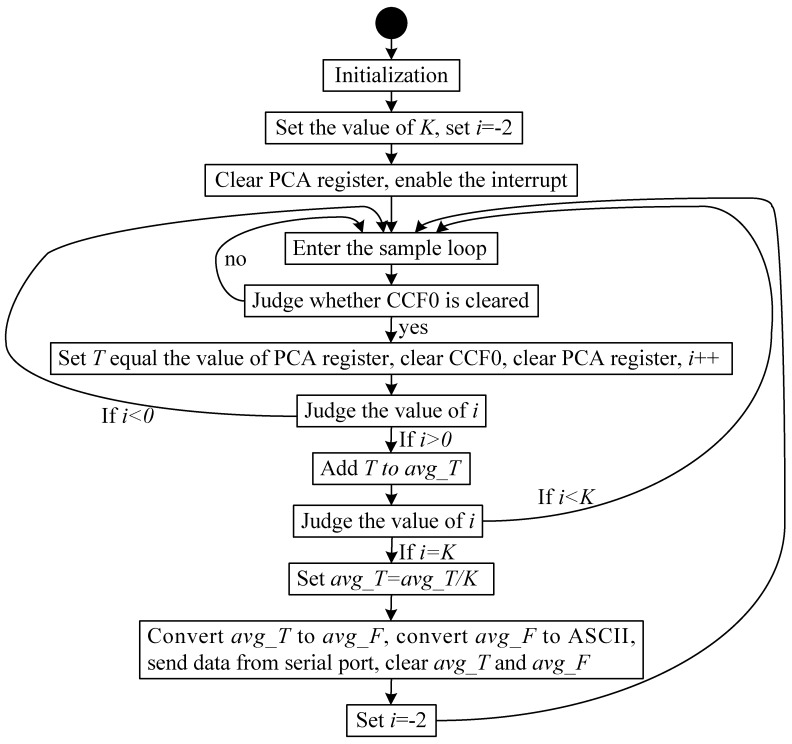
Workflow of frequency meter. PCA, programmable counter array.

The operations for converting the float-type variables to ASCII and sending data through the serial port would require several machine cycles, so the first and second cycles measured would be inaccurate. To omit the first two cycles, *i* is initialized as −2, and we provide that, if *i* < 0, the cycle data are abandoned. Figure 15 represents the workflow of the MCU. The Equation (31) can be considered as a function whose independent and dependent variables are z and $f_{measure}$, respectively. When the machine cycle z changes to Δz, the dependent variable $f_{measure}$ would change to $\Delta f_{measure}$. The relationship can be represented as follows:

(32) $$f_{measure}+\Delta f_{measure}=\frac{f_{osc}}{z+\Delta z}$$

The derivation of Equation (31) is as follows:

(33) $$\frac{df_{measure}}{dz}=-\frac{f_{osc}}{z^{2}}$$

This can also be represented as:

(34) $$df_{measure}=-\frac{f_{osc}}{z^{2}}dz$$

When dz=1 and T=0.1 ms we obtain the following equations

(35) $$z=T\times f_{osc}=10^{-4}\times 24.5\times 10^{6}=2450\;\mathrm{and}\;df_{measure}=-\frac{f_{osc}}{z^{2}}dz=-\frac{24.5\cdot 10^{6}}{2450^{2}}=4.0816\mathrm{Hz}$$

We find that, if z is larger, $df_{measure}$ would be smaller. That means that, if $f_{measure}$ is lower (the signal cycle is longer), the output frequency error of the program caused by measurement error would be reduced.

## 4. Experimental Section

### 4.1. Design of the Experimental Hardware Platform

**Figure 16:**
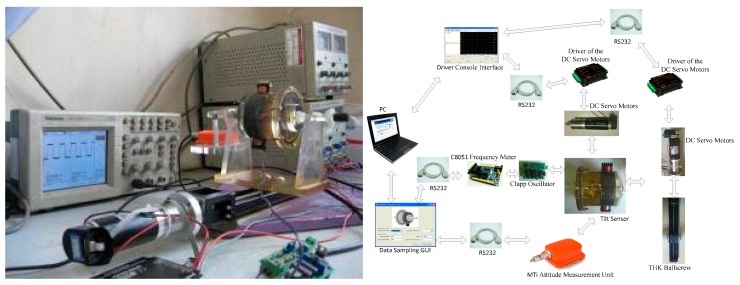
Hardware structure of the experimental platform.

The hardware structure of the experimental platform is shown in Figure 16. Changes in the sensor rotation angle cause changes of inductance that are measured as variations in the frequency of the oscillator circuit, as measured by the C8051 MCU and sent to the upper computer by the serial port. The program on the upper computer saves the frequency and the angle corresponding to the frequency. Here, we define the angle corresponding to the minimum frequency as 0°. Simultaneously, we correct the angle of the place where the MTi-28A sensor is fixed to make the roll angle measured by the MTi sensor output 0°. The rotation of the sensor is controlled by a DC motor. The speed of the DC motor is controlled by the upper computer through a driver. The sensor is fixed on the slider of a linear guide that controls the translational motion of the sensor. The slider’s moving direction and speed are controlled by another DC motor, which is controlled by a signal input by the upper computer through a driver. The specific settings of DC motors are shown in Appendix B.

The parameters of the tilt sensor are shown in Table 1, and we take the permeability of a vacuum $\mu_{0}$ for $4\pi \times 10^{-7}$ N/A^2^.

### 4.2. Design of Experimental Software Platform

**Figure 17:**
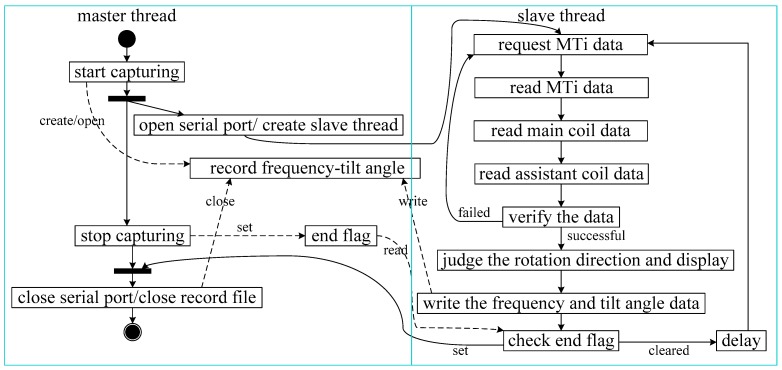
Activity diagram of frequency–roll angle synchronous collection program.

A frequency–roll angle synchronous collection program running on the upper computer is required to obtain the data from the sensor and the MTi-28A sensor. The program can be operated on a multithreading operating system. The collection program in this experiment was developed with C# and runs on the Microsoft. NET Framework platform. Figure 17 presents a unified modeling language (UML) activity diagram of the synchronous collection program. After program initiation, the program is divided into a master thread and a slave thread.

The main functions of the master thread concern interaction. These functions include the following: Create and open the frequency-roll angle record file after receiving the order to begin capture. Enable the serial port, create and start the slave thread. When the collection task of the slave thread starts to work, the master thread waits for the stop order. After receiving the order to stop data capture, the master thread stops setting the flag and waits for the slave thread to stop working. The serial port is then stopped and the record file closed.

The slave thread’s main function is synchronous data collection. After initiation, the slave thread progresses though a loop. In this loop, first, a request for data is sent to the MTi sensor, and the MTi sensor returns the rotation direction. The frequency data from both the main coil and the assistant coil are then separately obtained from the C8051 MCU via two serial ports, and the first valid piece of data in this sample period is selected. The direction returned from the MTi sensor is employed to verify the frequencies obtained from the MCU. If data are missing or the verification fails, the slave thread returns to the loop to obtain fresh data. If all data are correct, the frequencies of the main coil and the assistant coil are input to the module to determine the direction of rotation, and the result is displayed on the interface. The frequency and MTi sensor data are then written to the record file for analysis. At the end of the loop, if the end flag is set in reset mode, the slave thread continues to collect and record fresh data. If the flag is set to stop, the loop is broken, and the slave thread ceases operation.

Because the slave thread reads the MTi sensor data prior to reading the frequencies, the reading operations are not strictly synchronous. However, the time between the two operations is negligible, and we can consider the MTi sensor and frequency data as being synchronous. It is noteworthy that, if the delay of the slave thread loop is shorter than the time interval between the frequency return operations of the MCU, the returned frequency may be invalid.

The previous section introduced the method of determining the sensor’s angle of rotation direction using the assistant coil. This section describes the program for determining the direction of the sensor’s rotation in the experiment. 

When the main coil is at an angle of ±180° the method discussed is not available. However, if the sensor rotates sufficiently slowly, the rotation direction would be roughly equivalent to that obtained at the previous sampling period.

**Figure 18:**
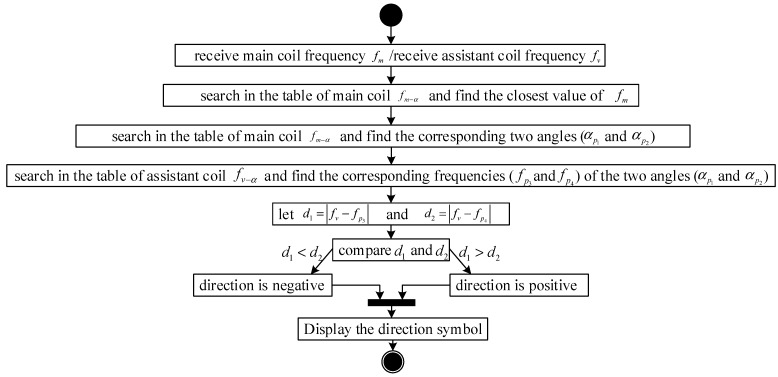
Unified modeling language (UML) activity diagram of rotation direction determination module.

The UML activity diagram in Figure 18 represents how the rotation direction determination module functions.

**Figure 19:**
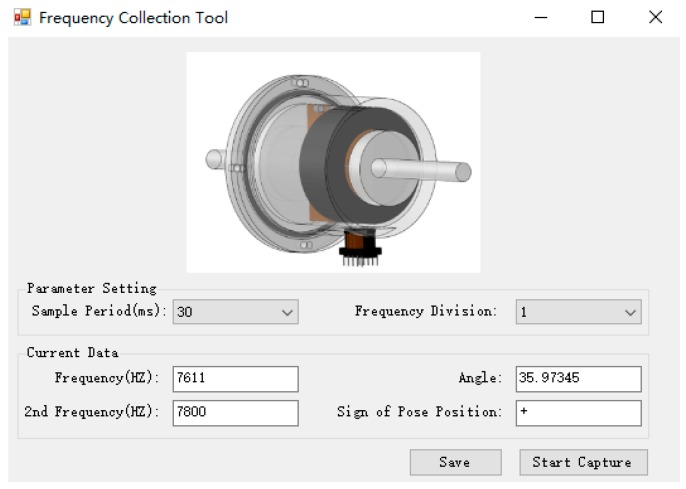
Graphical interface of frequency–roll angle synchronous collection program.

Figure 19 provides a screenshot of the graphical interface of the frequency-roll angle synchronous collection program, which represents the process of determining the rotation direction under the circumstance where the number of the coil turns is 1070, the frequency of the main coil $f_{m}$ is 7611 Hz, the frequency of the assistant coil $f_{v}$ is 7800 Hz, and the angle of the MTi sensor is 35.93°. Furthermore, $f_{p_{3}}$= 7639 Hz, $f_{p_{4}}$= 7798 Hz, $d_{1}>d_{2}$, and $f_{p_{4}}$ is more approximate to $f_{v}$, so that the sign of the direction is positive (+).

## 5. Results and Discussion

### 5.1. Experiments of the Sensor in Pure Rotation

We let the MTi sensor and our sensor be fixed together. While rotating these sensors, we let them do reciprocating translation along the horizontal line (translational motion is the interference of the experiments). In the process of motion, the output data of the MTi sensor and the frequency data of our sensor can be sampled. The output data of the MTi is the tilt angle. The data of the MTi are used as a benchmark. The output data of the MTi are taken as the x-axis and the primary frequency data of our sensor are taken as the y-axis. Then their corresponding relationships were plotted to compare and analyze the theoretical and experimental data.

**Figure 20:**
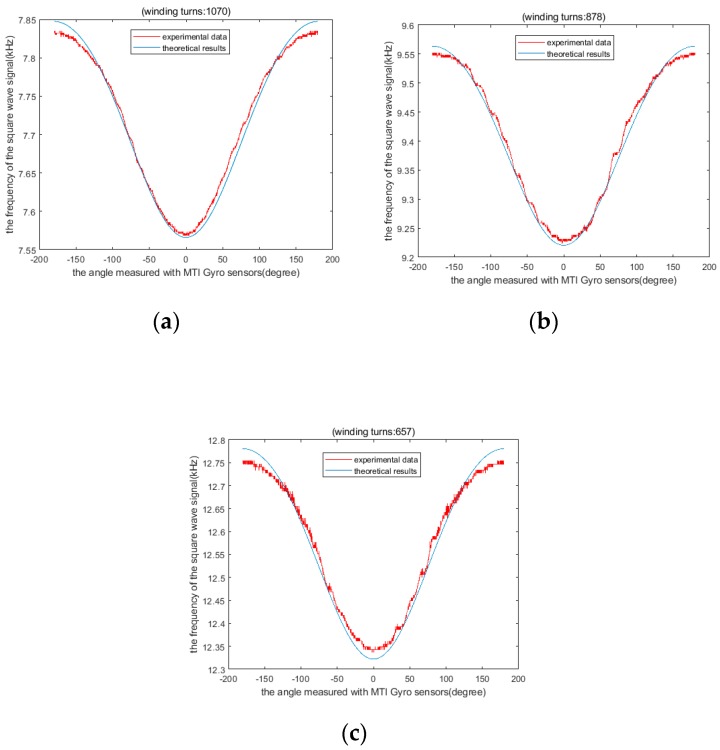
Comparison of theoretical results and experimental data: (**a**) 1070 turns, (**b**) 878 turns, (**c**) 657 turns.

Using four different numbers of turns of the inductor coil to obtain four inductors that have different values, sensor experiments were conducted to verify the above theoretical analysis. The sensor data with 1070-turn, 878-turn and 657-turn coils were compared with the theoretical calculation data, respectively. As they are separately shown in Figure 20a–c, respectively.

The output frequency of the sensor varies with the number of turns of the coil. On the one hand, we find that the experimental data of sensors with different turns of coils are basically consistent with the theoretical data. On the other hand, there is some error between the theoretical and experimental data due to the influence of sensor size parameters error and noise. 

Since the MTi sensor has excellent performance in both range and precision, we regard it as the calibration reference sensor of our sensor. In this way, we can get a comparison between the experimental curve and the theoretical calculation curve, as shown in Figure 20. The blue curve in Figure 20 is the theoretical curve drawn according to Equation (22). The red curve in Figure 20 is the curve drawn by the frequency and angle data of the sensor we designed based on the experimental measurement.

We can offer several explanations for the difference between experimental and theoretical results in Figure 20. For example, the coaxiality of the rotor inside the sensor will cause deformation of the experimental data curve. The buoyancy torque and gravity moment are not enough to balance the translational acceleration disturbance. That is the cause of the oscillation of sensor data when the sensor has translational motion. Because the coil is manually wound, there is an error in the number of turns of the coil. This causes deviation of the experimental data and theoretical curves in the y-axis direction.

### 5.2. Experiments of the Sensor in Rotation with Translational Interference

**Figure 21:**
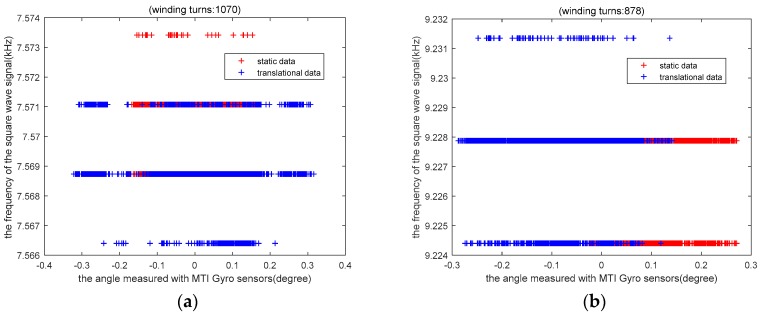
Comparison of static and translational data: (**a**) 1070 turns, (**b**) 878 turns.

For the sensor fixed with 878-turn and 1070-turn coils, the data are sampled under static and translational motion conditions, respectively. A comparison of static and translational data is separately shown in Figure 21a,b.

As can be observed from the figure, the frequency measurement error with the 1070-turn coil is within about 3 Hz, and the translational impact on the frequency error is less than about 5 Hz. The frequency measurement error with the 878-turn coil is within about 4 Hz, and the translational impact on the frequency error is less than about 8 Hz. In conclusion, the impact of the translational motion on sensor performance is relatively small. According to the results of the experiments, we find that the frequency of the coil with 1077 turns fluctuates within 5 Hz under the influence of translational noise and a zero tilt angle. Similarly, the output frequency of this coil with 1077 turns fluctuates within 8 Hz. According to the theoretical calculation based on Equation (22), under the condition of given frequency variation, the corresponding angle variation is calculated. Then we can get that the effect of 8 Hz frequency fluctuation on the output angle of the sensor is less than 1°. These results indicate that a measurement range of −180° to 180° was achieved, with an accuracy of 1°.

**Figure 22:**
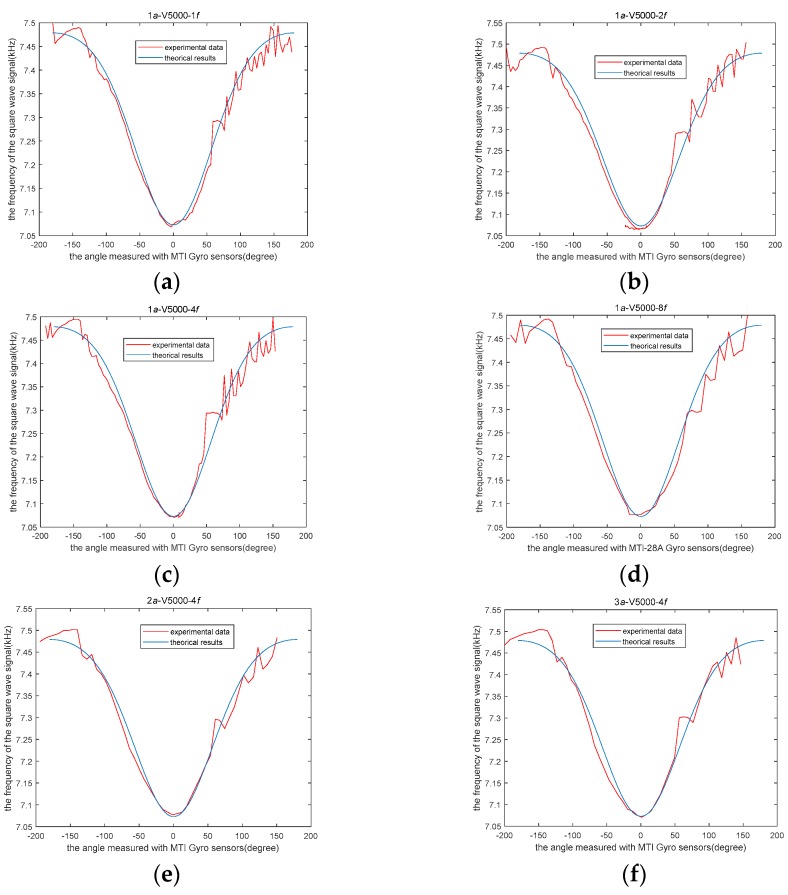
Comparison of theoretical results and experimental data with various amplitude and frequency of translational acceleration.

The experiments of the tilt sensor with rotation under the disturbance of translational motion with varying amplitude and frequency of the translational acceleration were achieved. The experimental parameters to describe the variations in amplitude and frequency of translational acceleration are shown in Table 2. The results of the experiments are shown in Figure 22a–f.

The experimental results show that translational noise had little effect on the characteristics of our sensor.

### 5.3. Experiments of the Sensor Compared with Three Other Sensors

We achieved some experiments of our sensor compared with three other sensors that are at the mature product level: MTi-28A, CXTA-01, and MPU6050. The four sensors were fixed together on the test platform, and the experiments were carried out in the condition of reciprocating translational motion with various amplitudes and frequencies of translational acceleration. 

**Figure 23:**
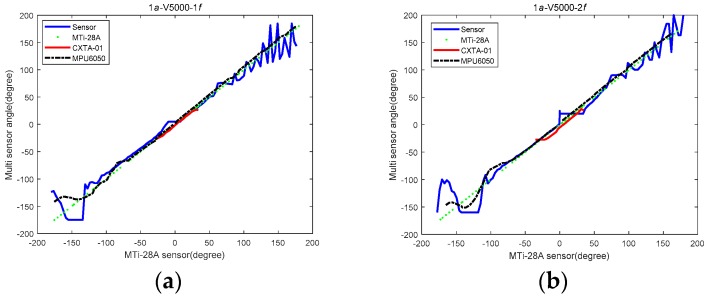

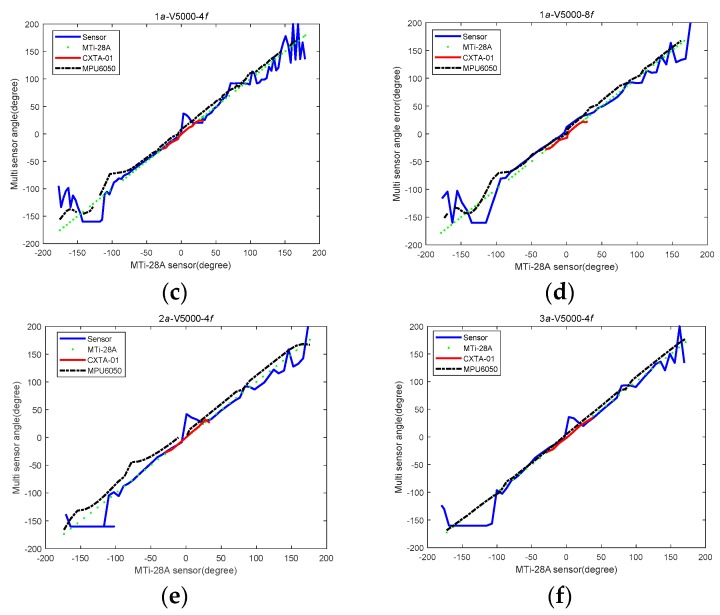
Comparison of the sensor and three other sensors.

The experimental parameters to describe the variations in amplitude and frequency of translational acceleration are shown in Table 2. The results of the experiments are shown in Figure 23a–f.

As the MTi sensor was the best in the measurement range and accuracy, we used it as the benchmark for comparison. Figure 23 shows the results of the comparative experiments, with MTi sensor data as the abscissa and angle data of the sensors as the ordinate under different amplitudes and frequencies of translational acceleration, where the data curve of the MTi sensor is the green curve whose equation is *y* = *x*.

It is shown that the other three sensors had some errors in addition to the MTi sensor as the benchmark. The experimental results show that the performance of our sensor was close to that of the three comparison sensors. It can be found that our sensor has some deviation from the experimental results. We can offer several explanations as to why the error exists. First of all, the manufacturing process of the sensor principle prototype made in our laboratory is not as good as that of the commercial sensors. Second, our sensor outputs raw data, which are not processed by algorithms such as the Kalman filter. In this case, the performance of our sensor can be close to or consistent with that of other sensors for comparison. It shows the effectiveness of our sensor.

As for the phenomenon that the data curve of our sensor has a small protrusion near the zero point in the figure, we can give the following two explanations. 

On the one hand, the change rate of the experimental data at the zero point is the smallest. The slope of the zero point of the theoretical curve is zero. The maximum value of the slope is obtained at about ±60°. As a result, the ability of our sensor to measure the dynamic tilt angle is the best at about ±60°. However, this ability is relatively poor at 0° and ±180°.

On the other hand, in the experiments of this section, our sensor and the three other sensors started to rotate from zero at the same time. When the rotational acceleration applied to the sensor is large enough, the torque of inertia acting on the damping board of the sensor will pull it away from the plumb direction. Especially when the buoyancy torque and gravity torque applied to the damping plate are not enough to offset the inertia torque, it will cause the damping plate to deviate from the plumb direction, resulting in a large error in the output data. Similarly, when the sensor is turned to ±180°, there will be rotational acceleration, first slowing down and then accelerating, which will make the damping plate oscillate away from the direction of the plumb. It is worth mentioning that the small bulge near the zero point in the curve of our sensor in the figure is mainly due to the applied rotational acceleration. The translational acceleration only plays a minor role.

### 5.4. Key Influencing Factors 

The key influencing factors are presented as follows:

(a) The larger the volume of the damping plate is within the allowable range of the structure, the greater the buoyancy obtained.
(b) The density distribution of the damping plate also affects the dynamic characteristics of the sensor. It determines both distance *l*_G_ and *l*_B,_ which affect the gravity torque and the buoyancy torque of the damping board, respectively. The larger the values of those two distances, the better the anti-interference ability of the sensor for translational noise.
(c) The density of liquid damping inside the sensor also affects the dynamic characteristics of the sensor.
(d) The system frequency *f*_osc_ and 16-bit PCA register of the C8051 single-chip microcomputer that was used to measure the oscillation frequency value of the sensor determine the accuracy of the sensor.

Equation (28) has no analytical solution, it only has numerical solution. The coefficients of the differential equation affect the maximum value of the numerical solution. If we take the coefficients of the differential equation as the independent variables and the absolute value of the maximum value of the angle in the numerical solution obtained by the differential equation after each set of coefficients is given as a dependent variable, then we can get a multivariate function as following:

(36) $$\left|\alpha_{\max}\right|=P(l_{G},l_{B},m,\rho ,V,a,f)$$

**Figure 24:**
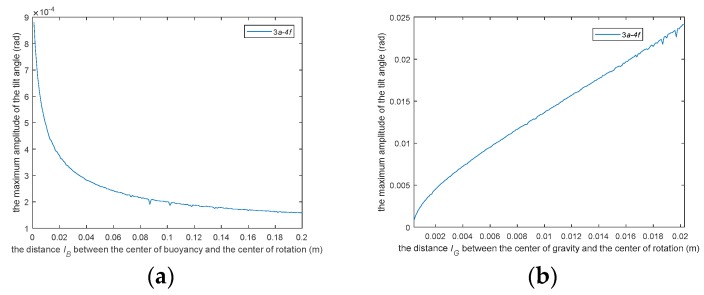

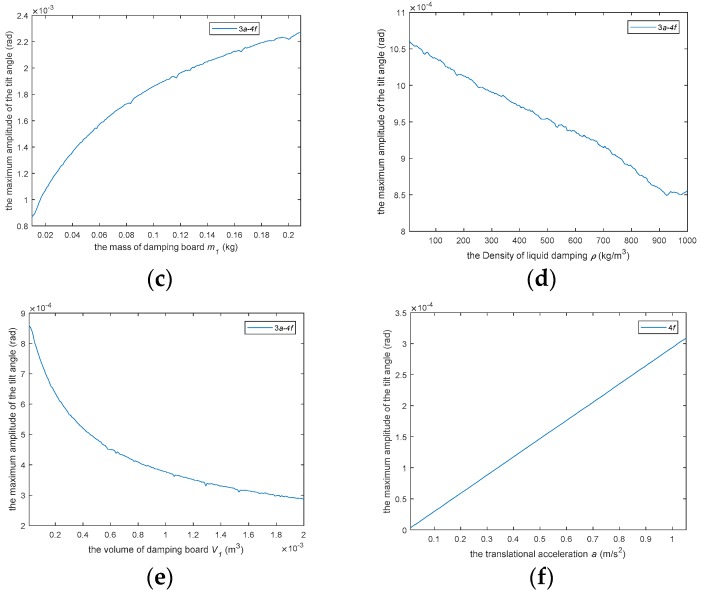
Key influencing factors of the sensor.

The coefficients of the Equation (28) are the parameters of the sensor. They affect the characteristics of our sensors. In order to show the influence of the parameters on sensor performance more intuitively, we give the other six fixed values of the independent variables. The function *P* is reduced to a univariate function:

(1) First, we analyzed the influence of parameter *l_B_* on the dynamic characteristics of the sensor. All the parameters except parameter *l_B_* were set to a fixed value respectively. We can get an unary function as shown in the Figure 24a below.
(2) Similarly, the influence of parameter *l_G_* on the dynamic performance of the sensor is shown in the Figure 24b below.
(3) The influence of the mass *m*_1_ of the damping board on the sensor is shown in the Figure 24c below.
(4) The effect of the density of the liquid damping on the characteristics of the sensor is shown in the Figure 24d.
(5) The effect of damper plate volume *V*_1_ on sensor characteristics is shown in the Figure 24e.
(6) The influence of translational acceleration *a* amplitude on sensor characteristics is shown in the Figure 24f.

Obviously, this is a multi-parameter optimization problem. In future work, we will carry out the optimization design of the parameters of the sensor.

Remark 1: This paper provides the first results on the design and implementation of a novel inclination sensor. This sensor can effectively reduce the influence of translation noise, and the measurement range of the sensor is larger than that of the sensor in [18].

Remark 2: Compared with the excellent commercial MTi sensor, the measurement error of the sensor comes from the fabrication process. The principle prototype in the laboratory is not elaborate enough. 

Remark 3: The sensor designed in this paper, according to the needs of different scenarios, can be made not only very large, but also very small. However, sensors with this structure can only measure the tilting angle of a single axis.

## 6. Conclusions 

A new dynamic tilt sensor is proposed in this paper. The measuring principle of the sensor is presented, and the design of its mechanical structure is described. In addition, a theoretical analysis of the sensor characteristics is provided. A physical prototype of the sensor was fabricated, and experiments of its performance were conducted on a test platform that provided both rotational and translational motion. The experimental data verified the validity of the theoretical analysis and the effectiveness of the designed sensor.

This study provides the following innovations:

(1) The structure of the dynamic tilt sensor is designed based on an eccentric principle, which has the advantage of a wide measuring range.
(2) The translational noise of the sensor is reduced by the internal damping effect of the liquid with which the sensor is filled.
(3) The translational noise of the sensor is also reduced by a design that places the center of gravity and the center of buoyancy at non-coinciding points.

Future work is as follows.

(a) We will carry out the multi-parameter optimization to refine the parameters of the sensor.
(b) The machining accuracy of sensor structure components needs to be further improved.
(c) The assembly accuracy of sensor structure parts needs to be further improved.
(d) Filtering algorithms should be developed for the sensor to process its output raw data.
(e) Research on the influence of viscosity coefficient on the characteristics of the sensor is needed in the future.
(f) Our own sensor calibration platform is not precise enough, there are calibration errors.

## Figures and Tables

**Table 1:** Parameters of the tilt sensor.

System Parameter	Specification
Number of turns(rotation)	1070
Number of turns(rotation) Number of turns(rotation) Number of turns(rotation & translation) Capacitance parameter *C*_1_ Capacitance parameter *C*_2_ Capacitance parameter *C*_3_ Radius *R* Radius *r* *a* *b*	878 657 1030 220 nF 220 nF 10 nF 32 mm 31.5 mm 6 mm 3 mm
*δ*(rotation) *δ*(rotation & translation)	0.18 mm 0.44 mm

**Table 2:** Experimental parameters of tilt sensor in translational motion with various amplitude and frequency of translational acceleration.

Results of Various *a* and *f*	Figure	Title
1*a* V5000 1*f* 1*a* V5000 2*f* 1*a* V5000 4*f* 1*a* V5000 8*f* 2*a* V5000 4*f*	Figure 22a Figure 22b Figure 22c Figure 22d Figure 22e	1*a*-V5000-1*f* 1*a*-V5000-2*f* 1*a*-V5000-4*f* 1*a*-V5000-8*f* 2*a*-V5000-4*f*
3*a* V5000 4*f*	Figure 22f	3*a*-V5000-4*f*

Note: *f* = 3.2546 Hz, *a* = *a_N_* = 0.1053 m/s^2^, V5000 = 0.0088 m/s.

## Appendix A

The permeance of all portions of the gaps shown in Figure 9 is presented in Table A1.

**Table A1:** Permeance of all portions of gaps shown in Figure 9.

Number	Portion of Gap	Permeance of Portion
1		$\Lambda_{1}=\frac{2\mu_{0}A}{d}=\frac{2\mu_{0}ab}{d}$
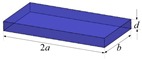
2		$\Lambda_{2}=\frac{\mu_{0}A}{d}=\frac{\mu_{0}ab}{d}$
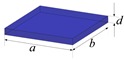
3		$\Lambda_{3}=\mu_{0}a$
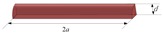
4		$\Lambda_{4}=0.5\mu_{0}b$
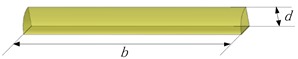
5		$\Lambda_{5}=0.5\mu_{0}a$
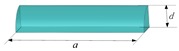
6		$\Lambda_{6}=0.308\mu_{0}d$
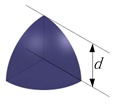
7		$\Lambda_{7}=\frac{4\mu_{0}a}{\pi }\ln(1+\frac{m}{d})\overset{m=d}{\to }\frac{4\mu_{0}a}{\pi }\ln(2)$
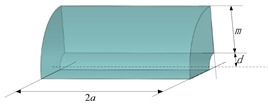
8		$\Lambda_{8}=\frac{2\mu_{0}b}{\pi }\ln(1+\frac{m}{d})\overset{m=d}{\to }\frac{2\mu_{0}b}{\pi }\ln(2)$
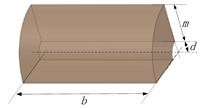
9		$\Lambda_{9}=\frac{2\mu_{0}a}{\pi }\ln(1+\frac{m}{d})\overset{m=d}{\to }\frac{2\mu_{0}a}{\pi }\ln(2)$
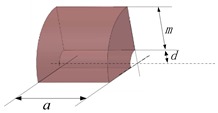
10		$\Lambda_{10}=\mu_{0}\frac{m}{2}=\mu_{0}\frac{d}{2}$
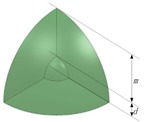

Based on Table A1, the reluctance of air gaps $R_{g1}$, $R_{g2}$, and $R_{g3}$ can be accurately expressed as follows:

$$R_{g1}=R_{g3}=\frac{1}{\Lambda_{2}+2\Lambda_{4}+2\Lambda_{5}+4\Lambda_{6}+2\Lambda_{8}+2\Lambda_{9}+4\Lambda_{10}},R_{g2}=\frac{1}{\Lambda_{1}+2\Lambda_{3}+2\Lambda_{4}+4\Lambda_{6}+2\Lambda_{7}+2\Lambda_{8}+4\Lambda_{10}}$$

## Appendix B

During the sensor translation experiment, the movement of the translational motor is linear, with a speed given as 5000 rpm. The reduction ratio of the motor’s reducer is 19 so that the sensor’s translational speed *v_n_* is given as follows:

$$v_{n}=\frac{5000}{19\times 60}\times 0.002=\frac{1}{114}\;m/s\;$$

When the amplitude of translational acceleration is given by 1000 round/s^2^, we can obtain the acceleration of the sensor as follows:

$$a_{n}=\frac{2}{19}\;m/s^{2}$$

During the sensor rotation experiment, the motion of the motor is at a constant speed with an amplitude of 200 rpm. The reduction ratio of the motor’s reducer is 24 so that the sensor’s angular velocity *ω**_s_* is given as follows.

$$\varpi_{s}=\frac{200\times 2\pi }{60}\times \frac{1}{24}=\frac{5\pi }{18}\;\mathrm{rad}/s$$
